# Identification and Expression Analysis of the *CHX* Gene Family in *Capsicum annuum* L.

**DOI:** 10.3390/biology15010037

**Published:** 2025-12-25

**Authors:** Jing Wang, Jiaxin Huang, Xu Jia, Yanping Liang

**Affiliations:** College of Horticulture, Shanxi Agricultural University, Jinzhong 030801, China; wangjing315@sxau.edu.cn (J.W.); 15541217978@163.com (J.H.); xxu_0259@163.com (X.J.)

**Keywords:** *Capsicum annuum* L., CHX, gene family identification, bioinformatics analysis, expression analysis

## Abstract

The cation/H^+^ exchanger (*CHX*) gene family plays a vital role in maintaining K^+^/Na^+^ homeostasis in plants, yet its functional characterization in pepper (*Capsicum annuum* L.) remains largely unexplored. To elucidate the potential roles of *CHX* genes in stress adaptation and development in pepper, a genome-wide identification and systematic analysis of this gene family was performed. Using a combination of Hidden Markov Model (HMM) searches, phylogenetic reconstruction, conserved motif and promoter analysis, and expression profiling across tissues and under multiple stress conditions, a total of 23 *CaCHX* genes were identified, which are unevenly distributed across 10 chromosomes and classified into 6 phylogenetic subfamilies. Expression profiling revealed that most *CaCHX* genes were highly expressed in flowers, suggesting their potential involvement in reproductive development, while only *CaCHX12* and *CaCHX17* were detected in leaves. Under treatments such as abscisic acid (ABA), gibberellic acid (GA), NaCl, and jasmonic acid (JA), *CaCHX1*, *CaCHX20*, and *CaCHX23* exhibited distinct temporal expression patterns, suggesting their involvement in hormone-mediated stress responses. This study provides the first comprehensive genomic and transcriptomic overview of the *CHX* family in pepper, offering novel insights into its regulatory roles in flower development and stress tolerance and, thus supplying valuable genetic resources for molecular breeding aimed at enhancing pepper resilience.

## Simple Summary

Pepper is an important vegetable crop, but its growth is often threatened by environmental stresses such as high soil salinity, which affects yield and quality. This study comprehensively identifies and analyzes the cation/H^+^ exchanger (*CHX*) gene family in pepper, which helps plants to maintain internal chemical balance under stress. Twenty-three *CHX* genes, which were unevenly distributed across ten chromosomes, were identified and classified into six groups. Expression analysis revealed that most of these genes are highly active in flowers, suggesting a role in flower development. Under salt and hormone treatments, genes such as *CaCHX1*, *CaCHX20*, and *CaCHX23* showed distinct expression patterns: *CaCHX1* was rapidly induced, *CaCHX20* was suppressed, and *CaCHX23* decreased initially but later increased. These findings suggest that *CHX* genes may help pepper to cope with stress and regulate reproductive growth. This study provides valuable gene resources for the future breeding of stress-resistant pepper varieties.

## 1. Introduction

*Capsicum annuum* L., a widely cultivated solanaceous crop around the world, is used not only as a fresh vegetable and seasoning spice but also as a raw material for processing in industries such as pharmaceuticals and cosmetics [1,2]. According to FAOSTAT [3], the global harvested area of pepper reached approximately 3.8 million hectares, with a total production of over 40 million tons, highlighting its significant role in global agriculture and food security. However, pepper cultivation is often affected by various abiotic stresses such as salt stress, drought, and high temperature, which constrain yield and quality improvement [4]. Therefore, studying the stress resistance of pepper is of great importance. Among these stresses, soil salinization is a major environmental factor restricting crop growth, development, and yield [5,6]. Under salt stress conditions, plants initially experience osmotic stress, impairing the uptake of water and mineral nutrients. Salt tolerance largely depends on a plant’s ability to re-establish and maintain ion homeostasis under stress conditions [7,8]. In this process, various transporter proteins play critical roles in regulating the Na^+^/K^+^ balance and pH homeostasis [9,10].

The cation/proton antiporter (CPA) represents an important class of primary monovalent cation transmembrane transporters. Its core function is to reduce Na influx, promote Na^+^ efflux and compartmentalization, and thereby maintain low cytoplasmic Na^+^ concentrations [11,12,13]. All members of this family contain a Na^+^/H^+^ exchanger domain of approximately 400 amino acids and are divided into two subfamilies, CPA1 and CPA2, based on phylogenetic relationships [14,15,16]. Among these, CPA1 primarily includes the Na^+^/H^+^ exchanger (NHX) family, which is involved in Na^+^ balance regulation, whereas CPA2 comprises the K^+^ efflux antiporter (KEA) and the cation/H^+^ exchanger (CHX) families [17,18]. The *CHX* family is unique to higher plants and includes numerous members. It plays a significant role in the plant salt stress response by regulating the Na^+^/K^+^ balance and intracellular pH homeostasis [19,20]. CHX proteins typically contain 10–12 transmembrane domains and localize to the plasma membrane, tonoplast, or endoplasmic reticulum membrane. Most *CHX* genes identified in plants encode proteins comprising approximately 800 amino acids [21]. Research has shown that the *CHX* family is widespread in plants. For instance, 28, 17, 18, and 16 members have been identified in *Arabidopsis thaliana* (L.) Heynh. [22], *Oryza sativa* L. [23], *Solanum lycopersicum* L. [24], and *Zea mays* L. [25], respectively. In *A. thaliana*, these 28 members are extensively involved in processes such as K^+^ transport, pH homeostasis maintenance, and floral organ development [22]. For example, *AtCHX14*, which is localized to the plasma membrane, regulates K^+^ redistribution in *Arabidopsis* [26]; *AtCHX17* functions as a K^+^ transporter, responds to low pH, salt stress, ABA, and helps to maintain cellular ion balance by regulating K^+^ uptake and vacuolar allocation [27,28,29]; and *AtCHX21* and *AtCHX23* play key roles in pollen tube guidance [30]. In wild soybean, *GsCHX19.3* mitigates ionic toxicity under saline–alkali stress by promoting K^+^ uptake and reducing Na^+^ uptake or enhancing its efflux [31]. Overexpression of *AtCHX24* accelerates leaf senescence, indicating its role in regulating this process [32]. Furthermore, the wheat *TaCHX* gene is highly expressed in spikes, suggesting its potential involvement in the development of reproductive organs [33]. Overall, the *CHX* gene family plays a crucial role in transmembrane proton and ion transport, and has been widely implicated in regulating plant growth, development, and stress responses [34].

While the *CHX* gene family has been characterized in several plant species, its role in pepper remains largely unexplored. Given that pepper is particularly sensitive to soil salinity and often cultivated in arid and semi-arid regions with increasing soil salinization, understanding the mechanisms underlying ion homeostasis is crucial. The *CHX* genes, as key regulators of K^+^/Na^+^ balance and pH homeostasis, may play a particularly vital role in pepper compared with other species due to its high sensitivity to salt stress and reliance on reproductive success under stress conditions.

Based on the whole-genome data of pepper and integrated *CHX* family information from *A. thaliana* and tomato, this study systematically identified the *CHX* gene family in pepper. We analyzed their physicochemical properties, gene structures, evolutionary relationships, chromosomal distribution, cis-acting elements, and collinearity, in addition to examining their expression patterns across different tissues and under various stress conditions in order to elucidate the functional characteristics of this family. This research aimed to reveal the mechanistic roles of pepper *CHX* genes in growth, development, and stress responses, thereby providing a theoretical basis and genetic resources for breeding new pepper varieties with high stress resistance.

## 2. Materials and Methods

### 2.1. Materials

Seeds of the heat-tolerant pepper line ‘17CL30’ [35] were surface-sterilized by soaking in warm water at 55 °C for 15 min, followed by germination at 28 °C until radicle emergence. Germinated seeds were sown and grown in a growth chamber under a 16 h light/8 h dark photoperiod, with day/night temperatures set at 28 °C and 24 °C, respectively.

At the six-true-leaf stage, plants were treated with abscisic acid (ABA, 30 μM), gibberellic acid (GA, 2 μM), NaCl (200 mM), and, jasmonic acid (JA, 10 μM), while plants under normal growth conditions served as the control. Leaf samples were collected at 1, 1.5, 3, 6, 12, and 24 h after treatment initiation, immediately frozen in liquid nitrogen, and stored at −80 °C. Each treatment included three biological replicates, with each replicate consisting of 10 uniformly growing plants.

### 2.2. Methods

#### 2.2.1. Identification of *CaCHX* Gene Family Members

The complete genomic data of pepper (‘Zunla-1’ v3.0) were downloaded from the PepperBase database (http://www.bioinformaticslab.cn/PepperBase/, accessed on 10 July 2025), and the CHX protein sequences of tomato and *Arabidopsis* were obtained from the NCBI database (https://www.ncbi.nlm.nih.gov/, accessed on 10 July 2025) to construct a local database. To comprehensively identify the members of the *CHX* gene family in pepper, three methods were employed for preliminary screening: (1) The first was a search using the Hidden Markov Model (HMM) profile (Accession: PF00999) for the CHX domain from Pfam (v38.0), (2) The second was a search with a custom HMM built using hmmbuild (HMMER v3.4) under the Ubuntu system (v1.68), as follows: First, a high-confidence seed alignment was created using 21 well-annotated CHX protein sequences from *Arabidopsi*, encompassing the conserved Na^+^/H^+^ exchanger domain. This alignment was used to build a profile HMM using hmmbuild (HMMER v3.4). The model’s quality was evaluated by searching against the source database to ensure that it could recover all seed sequences (E-value < 1 × 10^−10^). This custom model was then used to search the pepper proteome. (3) The third method was a homologous search using known *A. thaliana* CHX protein sequences as queries via the BLASTP program in the BioEdit (v7.0.5.3) software. The E-value threshold for all searches was set to 1 × 10^−5^. Finally, the preliminary results obtained from the three methods were integrated. Candidate members were subsequently validated for the presence of the characteristic domain using the SMART (http://smart.embl-heidelberg.de/, accessed on 11 July 2025) and CDD (https://www.ncbi.nlm.nih.gov/cdd/, accessed on 11 July 2025) databases. Only protein sequences containing the complete cation/H^+^ exchanger domain were retained and definitively identified as the final *CaCHX* family members.

#### 2.2.2. Physicochemical Property Analysis and Subcellular Localization Prediction of CaCHX Family Members

The physicochemical properties of the identified pepper CHX family members, including the number of amino acids, molecular weight, and isoelectric point, were analyzed using the ExPASy online platform (https://www.expasy.org/, accessed on 13 July 2025). Subcellular localization of these proteins was predicted with the Cell-PLoc 2.0 server (http://www.csbio.sjtu.edu.cn/bioinf/plant-multi/, accessed on 13 July 2025).

#### 2.2.3. Chromosomal Localization Analysis and Phylogenetic Tree Construction of the *CaCHX* Gene Family Members

The chromosomal distribution of pepper *CHX* genes was plotted using the MG2C online tool (http://mg2c.iask.in/mg2c_v2.0/, accessed on 14 July 2025), based on their physical location information. To elucidate the evolutionary relationships within the *CHX* gene family, multiple sequence alignment of CHX protein sequences from *A. thaliana*, tomato, and pepper was performed using ClustalX (v1.83). A phylogenetic tree was constructed with the maximum likelihood method in the MEGA-X (v10.2.6) software under default parameters. Finally, the resulting tree was refined and visualized using the iTOL online platform (https://itol.embl.de/, accessed on 14 July 2025).

#### 2.2.4. Analysis of Gene Structure, Conserved Motifs, and Promoter Cis-Acting Elements in CaCHX Family Members

The conserved motifs of *CaCHX* family members were analyzed using the MEME online tool (http://meme-suite.org/tools/meme, accessed on 15 July 2025), with the number of motifs set to 15. Concurrently, conserved protein domains of these members were identified using the NCBI Batch CD-Search tool (https://www.ncbi.nlm.nih.gov/Structure/bwrpsb/bwrpsb.cgi, accessed on 15 July 2025).

The promoter sequences of each *CaCHX* family member, defined as the 2000 bp region upstream of the transcription start site, were extracted using TBtools (v2.210). Subsequently, cis-acting elements in these promoter sequences were predicted based on the PlantCARE database (http://bioinformatics.psb.ugent.be/webtools/plantcare/html/, accessed on 20 July 2025). Finally, all prediction results were visualized using TBtools (v2.210).

#### 2.2.5. CaCHX Family Synteny Analysis

The “Advanced Circos” and “One Step MCScanX” plugins in the TBtools software (v2.210) were used to perform synteny analysis within the pepper species for the *CHX gene* family, as well as between pepper and other species.

#### 2.2.6. CaCHX Protein–Protein Interaction Network Analysis

The protein–protein interaction network for the CaCHX family members was constructed using the STRING database (https://string-db.org/, accessed on 18 July 2025).

#### 2.2.7. Analysis of *CaCHX* Gene Expression Patterns

Transcriptome datasets used for *CHX* expression profiling were retrieved from the publicly available PepperHub (http://pepperhub.hzau.edu.cn/, accessed on 10 September 2022). Briefly, samples were derived from the elite Chinese breeding line “6421” (*C. annuum*) grown under standard glasshouse conditions. A total of 63 organ-specific and 402 stress/hormone-treated samples (leaf and root, 40-day-old seedlings) were collected at 0, 0.5, 1, 3, 6, 12, and 24 h post-treatment in quadruplicate. Total RNA was isolated with TRIzol, and 150 bp paired-end libraries were sequenced on an Illumina HiSeq 4000 platform yielding ~41 million reads per library. Clean reads were aligned to the Zunla-1 reference genome using HISAT2, gene-level counts were obtained with HTSeq, and expression values were normalized as FPKM. Only *CHX* gene FPKM values were extracted for the present study. The data were organized using Excel and visualized with TBtools (v2.210).

#### 2.2.8. RT-qPCR Analysis

Total RNA was extracted from pepper leaves using Tiangen’s Polysaccharide & Polyphenol Plant Total RNA Extraction Kit. cDNA was synthesized via reverse transcription using Jinsha Biology’s UnionScript First Strand cDNA Synthesis Mix (with dsDNase) and stored at −20 °C for subsequent use. Primers (Supplementary Table S1) were designed with the online tool Primer3 Plus (https://www.primer3plus.com/, accessed on 20 September 2025), using *β-Actin* as the internal reference gene. *β-Actin* was selected based on its previously reported stability in pepper under stress conditions [35]. RT-qPCR was performed using Vazyme AceQ SYBR Green Master Mix. The 20 μL reaction system contained: 10 μL of Taq SYBR Green qPCR Premix, 0.4 μL each of forward and reverse primers, 2 μL of cDNA template, and 7.2 μL of ddH_2_O. The thermal cycling protocol consisted of pre-denaturation at 95 °C for 30 s, followed by 40 cycles of denaturation at 95 °C for 10 s and extension at 60 °C for 30 s. This experiment was conducted with three biological replicates, and each sample was analyzed with four technical replicates. Relative gene expression levels were calculated using the 2^–ΔΔCT^ method. Multiple comparisons between groups were analyzed using one-way ANOVA. Statistical analysis was carried out with Microsoft Excel 2019 and IBM SPSS Statistics 27, and graphs with significance markers were generated.

## 3. Results

### 3.1. Identification and Analysis of Physicochemical Properties of CaCHX Family Members

This study identified 23 *CaCHX* family members from the pepper genome, which were sequentially designated *CaCHX1* to *CaCHX23* according to their chromosomal locations (Supplementary Table S2). Physicochemical analysis showed that these CaCHX proteins contain 330–849 amino acids, with molecular weights ranging from 36.64 to 93.26 kDa (Supplementary Table S2). Their isoelectric points (pI) vary from 5.35 to 9.86, comprising 8 acidic and 15 basic proteins (Supplementary Table S2). The instability indices of the CaCHX proteins range from 31.24 to 47.48, with 15 proteins below 40 considered stable and 8 above 40 classified as unstable (Supplementary Table S2). The aliphatic indices, reflecting thermal stability, range between 100.55 and 128.73, suggesting that most members exhibit high thermal stability (Supplementary Table S2). In addition, all CaCHX proteins showed positive grand average of hydropathicity (GRAVY) values, indicating that they are hydrophobic (Supplementary Table S2). Subcellular localization predictions revealed distinct compartmentalization among CaCHX members, which may underlie their functional diversification: CaCHX4 is predicted to localize to the chloroplast and endoplasmic reticulum, suggesting potential roles in organellar pH or ion regulation; CaCHX18 is specifically targeted to the vacuole, implying a dedicated function in vacuolar ion sequestration; and the majority of members (21) are primarily localized to the plasma membrane and vacuole, consistent with canonical roles in transmembrane ion transport and cellular homeostasis (Supplementary Table S2).

### 3.2. Chromosomal Localization Analysis of CaCHX Genes

**Figure 1:**
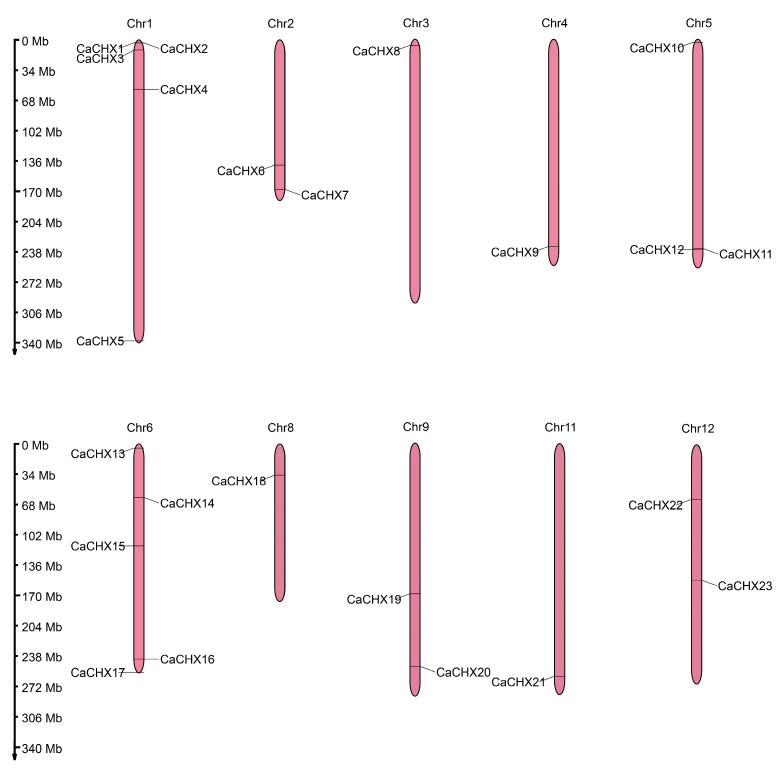
Chromosomal localization of the CHX genes in pepper.

The 23 *CaCHX* genes were unevenly distributed across 10 pepper chromosomes (Figure 1). Chromosomes 1 and 6 showed the highest distribution density, each containing five members, followed by chromosome 5 with three members. Chromosomes 2, 9, and 12 each contained two members, while chromosomes 3, 4, 8, and 11 contained only one member each.

### 3.3. Phylogenetic Analysis of Pepper CHX Genes

**Figure 2:**
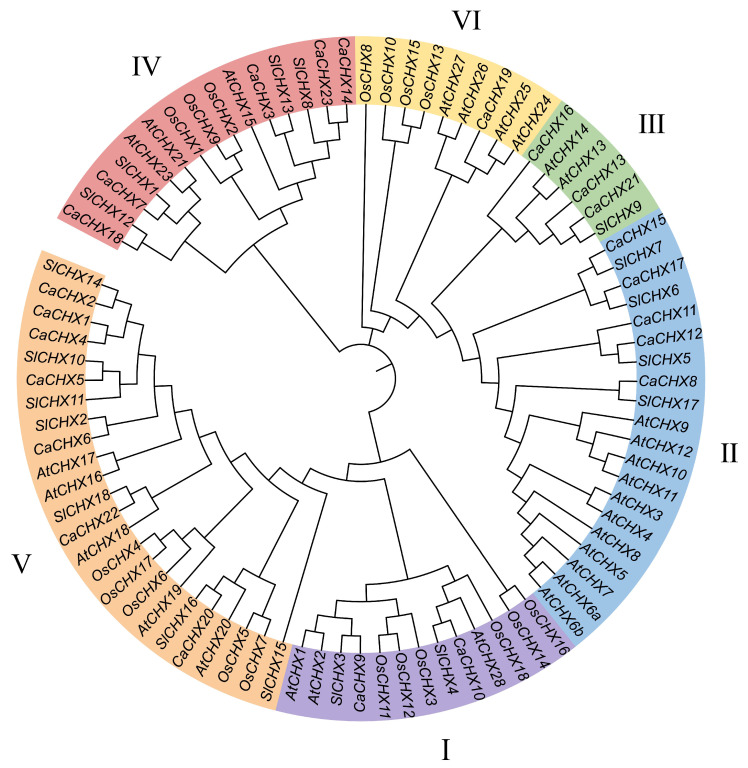
Phylogenetic tree of CHX family members of *A. thaliana* (*At*), *S. lycopersicum* (*Sl*), *O. sativa* (*Os*), and *C. annuum* (*Ca*).

A phylogenetic tree was constructed using CHX protein sequences from pepper (23), *Arabidopsis* (28), and rice (17) to elucidate the evolutionary relationships within the CHX family. Based on the clustering patterns observed in *Arabidopsis* and rice, the 68 proteins were classified into six subfamilies (I–VI) (Figure 2). The 23 CaCHX proteins were unevenly distributed among these subfamilies. Subfamily V contained the largest number of CaCHX proteins, with seven members, followed by subfamilies III and IV, each containing five CaCHX proteins. Subfamilies I and II contained two and three CaCHX proteins, respectively, while subfamily VI had the fewest members, with only one CaCHX protein (Figure 2).

### 3.4. Analysis of Conserved Motifs and Gene Structure

**Figure 3:**
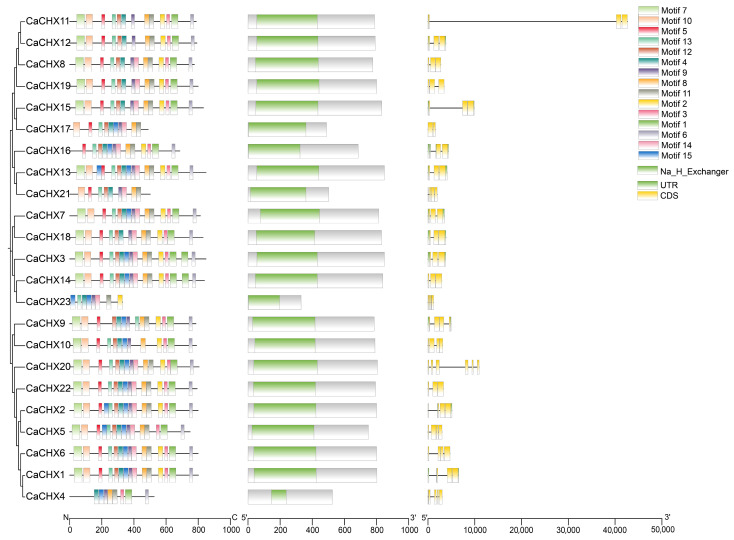
Prediction of CHX family protein structure in pepper.

Motif distribution analysis identified 15 conserved motifs among the 23 CHX proteins. All CaCHX proteins contained motif 4 and motif 9, with members of the same subfamily showing highly similar motif composition. Conserved domain analysis confirmed that all 23 CaCHX proteins possess the Na^+^/H^+^ exchanger domain. Gene structure analysis revealed that *CaCHX* genes contain 2–6 exons, with CaCHX1, 2, 3, 4, 7, 9, 12, 13, 15, 16, and 18 all containing untranslated regions flanking their coding sequences (Figure 3). The conserved motif analysis provides insights into the functional diversification and evolutionary relationships within the *CaCHX* family. The universal presence of motif 4 and motif 9 across all 23 members strongly suggests these motifs constitute the indispensable catalytic core or structural scaffold of the cation/H^+^ exchanger domain, a finding consistent with studies in *Arabidopsis* and rice [22,23]. Furthermore, the high similarity in motif composition among members of the same phylogenetic subfamily (Figure 3) reinforces the reliability of our subfamily classification (Figure 2) and implies that members within a subfamily may share redundant or overlapping biochemical functions.

Gene structure analysis revealed variation in exon count (2–6), which often correlates with functional complexity and regulatory potential in plant gene families. Interestingly, the presence of untranslated regions (UTRs) in 11 members (e.g., *CaCHX1*, *CaCHX4*, *CaCHX18*) suggests that these genes may be subject to more complex post-transcriptional regulation, potentially through mechanisms involving upstream open reading frames (uORFs) or miRNA binding sites. The structural diversity observed—both in conserved protein motifs and genomic architecture—parallels the functional specialization inferred from phylogenetic and expression analyses, supporting the hypothesis that the *CaCHX* family in pepper has evolved through a combination of sequence conservation in core functional domains and structural variation that may underlie regulatory and functional novelty.

### 3.5. Analysis of Cis-Acting Elements in CaCHX Family Members

**Figure 4:**
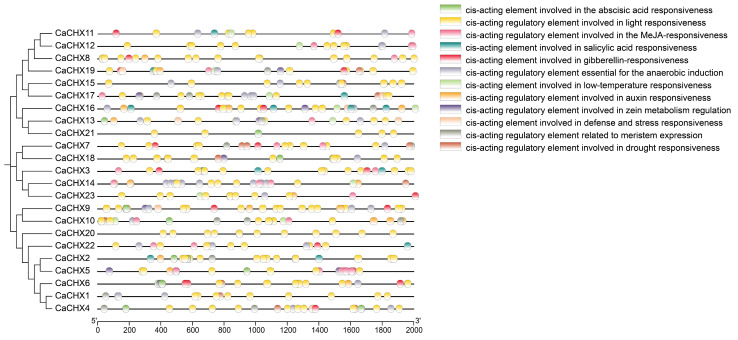
Analysis of cis-acting elements in the *CaCHX* promoters.

Analysis of the promoter regions of *CaCHX* genes identified various cis-acting elements, including those responsive to abscisic acid, light, methyl jasmonate, salicylic acid, gibberellin, anaerobic induction, low temperature, auxin, zeatin metabolism regulation, defense and stress responses, meristem expression, and drought. Among these, light-responsive elements were most abundant. The enrichment of these cis-regulatory elements suggests that pepper *CHX* genes are involved in plant responses to multiple pathogens and environmental stresses (Figure 4).

### 3.6. Synteny Analysis of the CaCHX Family

**Figure 5:**
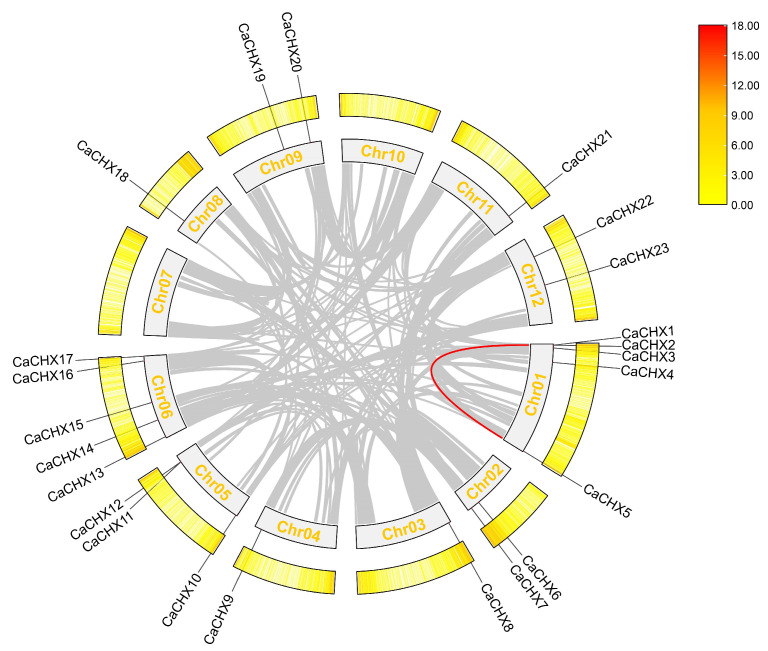
Inter-synteny analysis of *CaCHX*. *Circos plot structure:* Innermost ring (Chr00–12) represents chromosomes; yellow band indicates gene density; outermost ring shows chromosomal positions of 23 *CaCHX* genes. *Collinearity relationships:* Gray lines denote genome-wide duplication events; red lines indicate *CaCHX*-specific duplication events.

**Figure 6:**
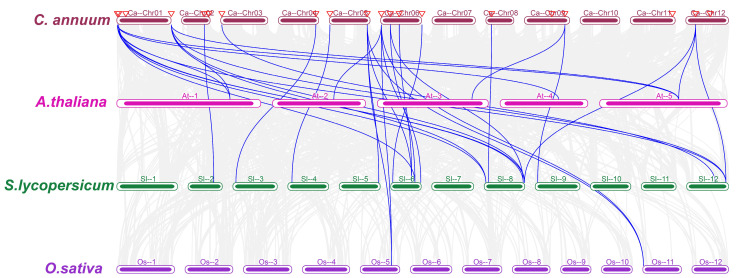
Synteny analyses of the *CHX* genes between *C. annuum* and three representative plants (*A. thaliana*, *S. lycopersicum,* and *O. sativa*) Gray background lines indicate synteny blocks across all genomes. Blue lines highlight syntenic *C. annuum CHX* gene pairs with *A. thaliana*, *S. lycopersicum*, and *O. sativa*. The red triangles indicate the *CHX* genes in pepper.

Intra-genomic synteny analysis revealed only one pair of syntenic genes within the pepper genome, specifically between CaCHX1 and CaCHX5 (Figure 5). Inter-species synteny analysis showed that pepper and tomato exhibited the highest degree of homology, with 23 syntenic gene pairs identified. By comparison, eight syntenic pairs were found between pepper and *A. thaliana*, while only four were detected between pepper and rice, representing the lowest number among the analyzed species (Figure 6).

### 3.7. Protein–Protein Interaction Network Analysis of the CaCHX Family

**Figure 7:**
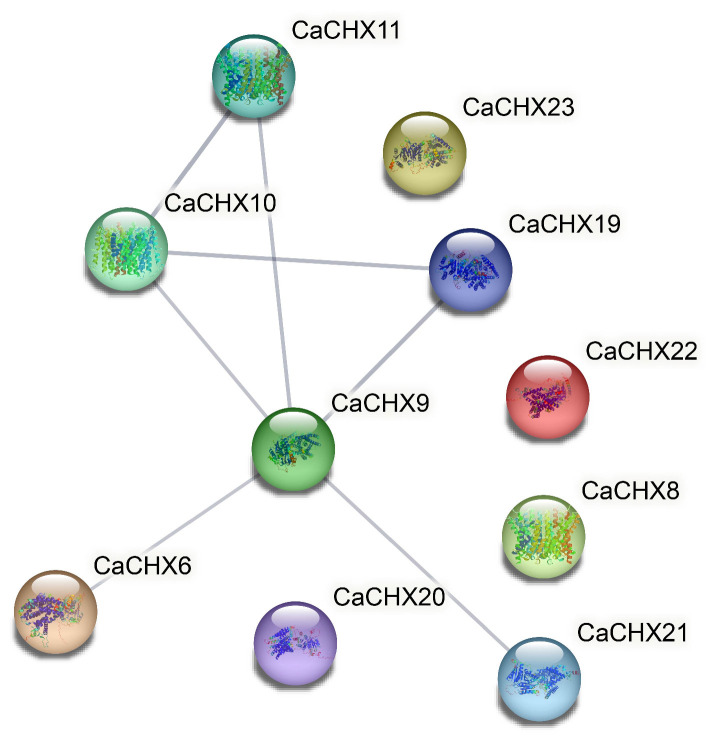
Protein interactions network among members of the CaCHX family. Nodes represent gene products, with color intensity indicating interaction degree (darker = higher connectivity). Line thickness corresponds to interaction strength (thicker = stronger associations). Edge saturation scales with confidence scores.

Protein–protein interaction analysis among CaCHX family members revealed interactions involving CaCHX6, CaCHX9, CaCHX10, CaCHX11, CaCHX19, and CaCHX21. Among these, CaCHX9 displayed the most extensive interactions, with five connecting partners (Figure 7).

### 3.8. Expression Analysis of the CaCHX Family in Different Pepper Tissues and Under Various Treatments

**Figure 8:**
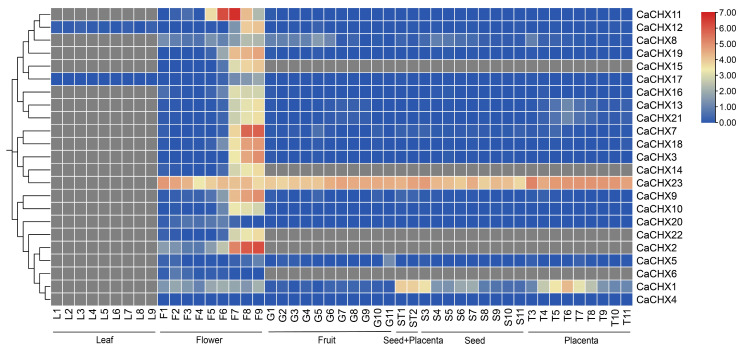
Expression analysis of the pepper *CHX* gene family in different tissues. L: leaf; F: f lower; G: fruit; ST: seed and placenta; S: seed; T: placenta. Gray color indicates no detectable gene expression.

Given the close relationship between gene expression levels and gene function, transcriptome data were used to analyze the expression levels of 23 *CaCHX* genes in pepper leaves, flowers, fruits, seeds, and placentas. The expression data were log_2_-transformed and normalized by Z-score on a row-wise basis. The results revealed distinct tissue-specific expression patterns among the *CaCHX* genes. As shown in Figure 8, the expression levels are represented as Z-scores (row-normalized). Here ‘high expression’ refers to cells with deep red color (Z-score > 1.5), indicating expression substantially above the gene’s mean across all tissues. Using this criterion, the vast majority of *CaCHX* members —such as *CaCHX2*, *6*, *14*, *15*, and *22*—were specifically or highly expressed in floral tissues. *CaCHX1* showed relatively high expression in seeds and placentas, while *CaCHX23* was highly expressed in all tissues except leaves. In leaf tissues, only *CaCHX12* and *17* were expressed (Figure 8).

**Figure 9:**
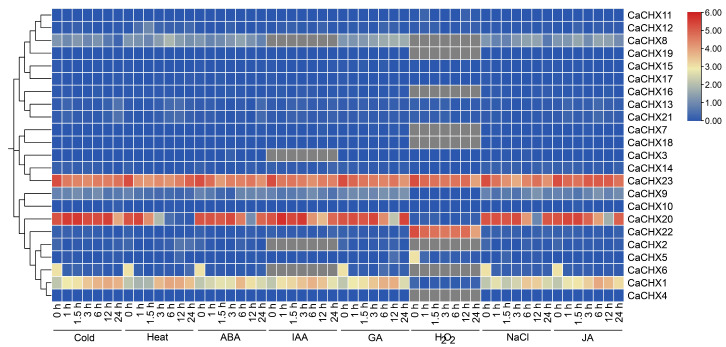
Expression analysis of the pepper *CHX* gene family under different stresses. Gray color indicates no detectable gene expression.

Based on transcriptome data, the expression patterns of *CaCHX* genes under various abiotic stress treatments (Cold, Heat, ABA, IAA, GA, H_2_O_2_, NaCl, JA) were analyzed. The results indicated that *CaCHX23* was highly expressed under all stress conditions. *CaCHX1* and *CaCHX20* exhibited relatively high expression under most stresses but were suppressed after H_2_O_2_ treatment. In contrast, *CaCHX22* was specifically and highly expressed only under H_2_O_2_ stress. The remaining members of the *CaCHX* family showed low expression across all stress conditions (Figure 9).

### 3.9. RT-qPCR Validation of Selected CaCHX Genes

To validate the transcriptomic trends and investigate dynamic responses, three candidate genes (*CaCHX1*, *CaCHX20*, and *CaCHX23*) were selected for RT-qPCR analysis under ABA, GA, NaCl, and JA treatments. We focused on expression changes that were both statistically significant (*p* < 0.05, one-way ANOVA with Tukey’s test) and exhibited a ≥2-fold difference, a commonly accepted threshold for biologically meaningful regulation in plant stress responses.

**Figure 10:**
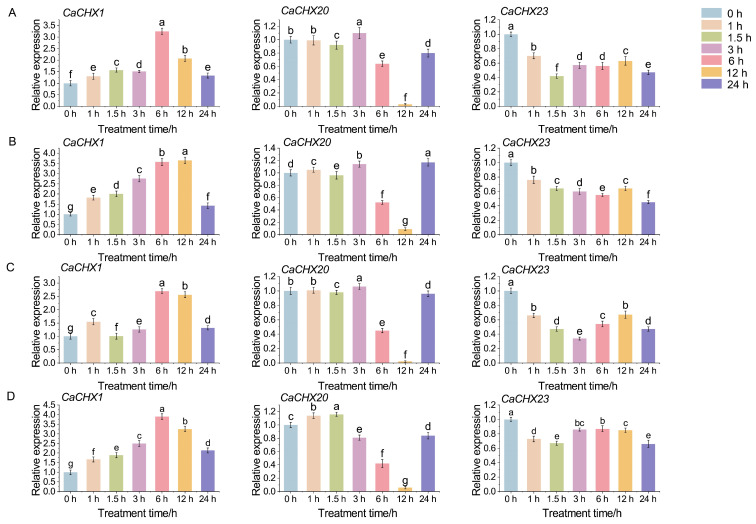
Relative expression levels of *CaCHX1*/*20*/*23* under ABA (**A**), GA (**B**), NaCl (**C**), and JA (**D**) treatments. Different lowercase letters indicate statistically significant differences at the level of *p* < 0.05.

The results revealed distinct temporal expression patterns (Figure 10). *CaCHX1* showed a strong and rapid induction, with expression levels increasing by more than 4-fold at 6 h post-treatment (hpt) under NaCl and JA, meeting our criteria for biologically significant up-regulation. In contrast, the expression of *CaCHX20* was significantly suppressed, showing a greater than 2-fold decrease by 12 hpt under most treatments, indicating a consistent down-regulatory response. *CaCHX23* exhibited a more complex pattern, with an initial decrease followed by a recovery or increase at later time points; however, the magnitude of change for *CaCHX23* largely remained below the 2-fold threshold at most time points, suggesting its role may be more modulatory than strongly inducible. The differential regulation of these genes underscores their potential distinct functions in mediating pepper’s response to hormonal and abiotic stresses.

## 4. Discussion

Pepper is an important vegetable crop, but it is susceptible to various biotic and abiotic stresses affecting both its yield and quality. Previous studies have shown that the *CHX* gene family is involved in floral organ development and ion balance regulation, playing a significant role in plant growth and stress responses [36]. Although *CHX* genes have been extensively studied in plants such as *A. thaliana*, tomato, rice, and maize, their functions in pepper remain unclear. Therefore, this study identified members of the *CHX* gene family in pepper, systematically analyzed their physicochemical properties, evolutionary relationships, promoter cis-acting elements, and synteny, and investigated their expression patterns across different tissues, under stress conditions, and in response to hormone treatments.

Through bioinformatics approaches, this study identified a total of 23 CHX family genes in pepper. Compared to *Arabidopsis* (28) and tomato (18), pepper possesses 23 *CHX* genes, suggesting a moderate expansion within the Solanaceae lineage [24]. Physicochemical property analysis revealed that most CaCHX proteins are stable alkaline proteins, and all CaCHX family members are hydrophobic proteins. Subcellular localization predictions indicated that CaCHX proteins are primarily distributed in the cell membrane and vacuole, consistent with their putative roles in transmembrane ion transport and vacuolar compartmentalization [37]. For instance, membrane-localized members such as CaCHX1 and CaCHX20 may mediate Na^+^ efflux or K^+^ uptake under salt stress, while vacuolar-localized CaCHX18 could contribute to Na^+^ sequestration, collectively enhancing cellular ion homeostasis.

Chromosomal localization analysis showed that the 23 *CaCHX* family genes are unevenly distributed across 10 pepper chromosomes, with notable clusters on chromosomes 1 and 6 (Figure 1). Such clustering, often observed in gene families expanded through tandem duplication, suggests potential functional redundancy or subfunctionalization among closely related members. However, synteny analysis revealed only one duplicated pair (*CaCHX1*/*CaCHX5*), and their divergent expression patterns under stress (Figure 9 and Figure 10) imply neofunctionalization rather than simple redundancy. This limited duplication contrasts with the extensive synteny observed between pepper and tomato (23 gene pairs), highlighting evolutionary conservation within Solanaceae but also indicating species-specific diversification of *CHX* genes.

To further analyze the phylogenetic relationships of pepper CHX family proteins, a phylogenetic tree was constructed using CHX proteins from *A.thaliana* (28), rice (17), and pepper (23). The results indicate that subfamilies III and V are particularly enriched in pepper, implying lineage-specific gene retention following whole-genome duplication events (Figure 2). Notably, the loss of certain CHX orthologs in pepper (e.g., AtCHX14-like genes) may reflect adaptation to distinct environmental niches. This widespread distribution suggests functional diversification early in plant evolution, with each subfamily potentially specializing in distinct physiological roles such as K^+^ transport, pH regulation, or reproductive development [11]. Promoter analysis revealed abundant cis-acting elements related to light, hormones (ABA, GA, JA), and stress responses (Figure 4), supporting the notion that *CaCHX* genes are integrated into complex signaling networks that coordinate development and stress adaptation.

Expression profiling further supports species-specific adaptations: while *Arabidopsis CHX* genes are broadly expressed in vegetative tissues, pepper *CHX* members show pronounced floral specificity, suggesting a functional shift toward reproductive development under stress-prone cultivation conditions. These expression divergences highlight the unique trajectory of *CHX* family evolution in pepper and its potential role in adapting to arid and saline environments. Most *CaCHX* members showed no or minimal expression in leaves, with only *CaCHX12* and *CaCHX17* being detected. This strong floral bias suggests that the *CaCHX* family may have undergone functional specialization towards reproductive processes in pepper, possibly diverting from ancestral roles in vegetative ion homeostasis. Notably, *CaCHX* genes were consistently highly expressed in floral organs, a characteristic that has also been reported in other species. For instance, *GmCHX15c* in soybean has been confirmed to be highly expressed in flowers [38], and *PbrCHX16* plays an important role in pollen tube growth of pear [39], implying a fundamental and conserved mechanistic role for *CHX* transporters in plant reproduction. We hypothesize that specific *CaCHX* members (e.g., *CaCHX2*, *6*, *14*, *15*, *22*) may regulate ion gradients (e.g., K^+^, H^+^) or pH within specific floral compartments, thereby influencing critical processes such as pollen tube guidance, stigma receptivity, or ovule development, analogous to the roles of AtCHX21/23 in *Arabidopsis* [30]. The findings of this study further support the potentially conserved function of the *CHX* gene family in reproductive development. The unique tissue-specific expression patterns of *CaCHX* genes suggest that different *CaCHX* members may mediate distinct biological processes in response to developmental signals and environmental stresses.

Numerous studies have demonstrated that the *CHX* gene family responds to abiotic stresses such as salt, drought, and low temperature [40]. The underlying mechanisms are diverse. For example, soybean *GmCHX1* enhances salt tolerance and stabilizes yield by promoting Na^+^ efflux [41], and it may synergize with *GmCHX20a* to counteract osmotic and ionic stress induced by high salinity [42]. In rice, *OsCHX14*, regulated by the JA signaling pathway, can transport K^+^, Rb^+^, and Cs^+^ in vivo and plays a critical role in maintaining potassium homeostasis during the heading stage [23]. Furthermore, heterologous overexpression of *KvCHX* from the coastal plant Anemone can significantly enhance salt tolerance in *Arabidopsis* seedlings [43]. A strong and rapid induction of *CaCHX1* was observed under diverse treatments including ABA, GA, JA, and NaCl (Figure 9 and Figure 10). This multi-stimuli responsiveness suggests that *CaCHX1* may function as a key integrator node, converging signals from distinct hormonal and environmental pathways. We propose several non-exclusive mechanistic explanations for this phenomenon. First, the promoter region of *CaCHX1* is enriched with various hormone- and stress-responsive cis-elements (Figure 4), which may allow it to be directly transcriptionally activated by different transcription factors downstream of ABA, GA, and JA signaling. Second, these disparate treatments may ultimately converge on a common cellular disturbance, such as disruption of K^+^/Na^+^ homeostasis or cytosolic pH fluctuations. As a putative cation/H^+^ exchanger, *CaCHX1* induction could represent a compensatory mechanism to re-establish cellular ion balance, a core function conserved within the CHX family [19,21]. Third, complex hormonal crosstalk may be involved; for instance, JA application might potentiate the ABA signaling pathway, leading to indirect *CaCHX1* activation. This multifunctional induction pattern positions *CaCHX1* as a prime candidate for mediating pepper’s adaptation to a broad spectrum of abiotic challenges. Future studies, such as promoter deletion analysis and electrophoretic mobility shift assays (EMSA), are needed to identify the precise transcription factors involved, and genetic manipulation of *CaCHX1* will clarify its functional necessity in this integrative stress response.

The widespread and sustained expression of *CaCHX23* under diverse stresses (Figure 9) suggests it may serve as a constitutive stabilizer of ion and pH balance. Its promoter contains multiple stress-responsive elements, which may allow it to be activated through several signaling pathways. Given its phylogenetic proximity to known pollen-specific *CHX* genes, CaCHX23 may also play a conserved role in reproductive development under stress, ensuring successful fertilization under adverse conditions.

In summary, the diversification of the *CHX* gene family in pepper appears to be driven by a combination of whole-genome duplication, tandem duplication, and functional specialization. While some members (e.g., *CaCHX1*) have evolved stress-responsive roles, others (e.g., *CaCHX23*) may retain broad housekeeping functions. The high expression in flowers underscores the importance of this family in reproductive success, possibly linking environmental adaptation to fertility. *CaCHX1* is hypothesized as a rapid stress-responsive regulator that may mediate Na^+^ efflux/K^+^ influx under salt and hormone treatments. *CaCHX23* likely serves as a constitutive ion homeostasis maintainer, especially in reproductive tissues.

While this study provides a comprehensive genomic and expression atlas of the *CHX* family in pepper, the functional roles of individual members—particularly *CaCHX1* and *CaCHX23*—remain to be experimentally validated. Future work should prioritize functional characterization via transgenic approaches, subcellular localization assays, and ion transport assays in heterologous systems to determine whether *CaCHX1* and *CaCHX23* directly mediate K^+^/Na^+^ homeostasis or pH regulation. Additionally, promoter-reporter assays and electrophoretic mobility shift assays are needed to confirm the in silico-predicted cis-regulatory elements and transcription factor binding sites.

## 5. Conclusions

In this study, 23 CHX family members were identified from the pepper genome, which were classified into 6 subfamilies and found to be unevenly distributed across 10 chromosomes. Prediction of promoter cis-acting elements indicated that *CaCHX* genes are primarily regulated by light and hormones. Expression analysis revealed tissue-specific patterns for *CaCHX* genes, with predominant high expression in floral organs, implying their potential involvement in the development of pepper reproductive tissues. The findings and hypotheses generated here—particularly regarding the roles of *CaCHX1* and *CaCHX23*—establish an essential foundation and directly inform the design of future functional studies. Subsequent work will focus on in vivo validation using transgenic approaches, such as subcellular localization, protein interaction assays, and functional characterization under stress conditions.

## Data Availability

The data used for the analysis in this study are available in the article and the Supplementary Materials.

## Supplementary Materials

The following supporting information can be downloaded at: https://www.mdpi.com/article/10.3390/biology15010037/s1, Table S1: RT-qPCR primers for *CHX* gene family; Table S2: Physicochemical properties of the CHX family in pepper.

## Abbreviations

The following abbreviations are used in this manuscript:

CHX	cation/H^+^ exchanger
CPA	cation/proton antiporter
NHX	Na^+^/H^+^ exchanger
KEA	K^+^ efflux antiporter
hpt	hours post-treatment
ABA	abscisic acid
GA	gibberellic acid
JA	jasmonic acid
